# First cases of contagious ovine digital dermatitis in Germany

**DOI:** 10.1186/s13028-020-00544-0

**Published:** 2020-08-27

**Authors:** Philip Christian Tegtmeyer, Gareth James Staton, Nicholas James Evans, Judith Rohde, Teresa Maria Punsmann, Martin Ganter

**Affiliations:** 1 Clinic for Swine, Small Ruminants and Forensic Medicine, University of Veterinary Medicine Hannover, Foundation, Bischofsholer Damm 15, 30173 Hannover, Germany; 2 Department of Infection Biology and Microbiomes, Institute of Infection, Veterinary and Ecological Sciences, University of Liverpool, Leahurst Campus, Chester High Rd, Neston, Liverpool, CH64 7TE UK; 3 Institute for Microbiology, University of Veterinary Medicine, Hannover, Foundation, Bischofsholer Damm 15, 30173 Hannover, Germany

**Keywords:** Footrot, Lameness, Sheep, *Treponema*

## Abstract

Contagious ovine digital dermatitis (CODD) is a significant disease of the ovine foot characterised by severe lameness and progressive separation of the hoof horn capsule from the underlying tissue. Similar to bovine digital dermatitis (BDD), pathogenic members of the genus *Treponema* including the *Treponema medium* phylogroup, *Treponema phagedenis* phylogroup and *Treponema pedis* are frequently found together in CODD lesions. To date, CODD was only described in Ireland and the United Kingdom. In northern Germany, cases of an unusually severe lameness presented in a sheep flock that had been affected by footrot for several years. These cases were non-responsive to conventional footrot therapies, with some sheep exhibiting substantial lesions of the claw horn that resulted in horn detachment. Lesion swab samples were collected from both clinically affected and asymptomatic animals. In all clinically affected sheep, CODD-associated *Treponema* phylogroups were detected by polymerase chain reaction. This is the first report of CODD in Germany and mainland Europe, indicating a wider geographic spread than previously considered. In cases of severe lameness attributed to claw lesions in sheep that fail to respond to footrot treatment, CODD should be considered irrespective of geographic location.

## Background

A novel disease of sheep claws, termed contagious ovine digital dermatitis (CODD), was first described in the UK in 1997 [1]. The disease is characterised by severe lameness associated with initial inflammation of the coronary band. The disease usually begins with an infection of the claw horn at the coronet, which underruns the hoof horn capsule abaxially. Subsequently, there is a progressive separation of the claw from the underlying tissue. In severe cases, the entire claw horn may be avulsed, leaving the sensitive lamellae exposed [1–5]. The consequences of the severe lameness include poor body condition and frequent recumbency [1, 4, 6]. In sheep, other causes of infectious lameness, including footrot and interdigital dermatitis, are frequently diagnosed. Ovine footrot, is considered to be caused by *Dichelobacter nodosus* and *Fusobacterium necrophorum*, and is strongly associated with CODD [7]. However, despite the detection of *D. nodosus* and *F. necrophorum* in CODD [2, 8], the disease is regarded as distinct from footrot or the associated condition, ovine interdigital dermatitis [9]. Whilst considered multifactorial and polymicrobial, there is substantial evidence that the major aetiological agents of CODD are *Treponema* spp., with implicated phylogroups identical to those involved in bovine digital dermatitis (BDD): *Treponema medium* phylogroup, *Treponema phagedenis* phylogroup and *Treponema pedis* [10–16]. Other *Treponema* -associated diseases with distinct clinical presentations have also been identified in goats [17–19] and wild elk [20].

CODD is now common across the United Kingdom. The proportion of farms affected by CODD ranges from 53% in England, with an intra-flock prevalence of 2.4% [21], to 35.0% in Wales, with median on farm prevalence of 2.0% [22]. In a recent follow-up survey of 1260 farmers in England, 48.7% of respondents reported CODD on their farms with a mean farm prevalence of 2.3% [23]. Until now, CODD has only been reported in the United Kingdom and Ireland, but it is likely that the disease has spread beyond these boundaries [24]. This report describes the first known outbreak of CODD in Germany and mainland Europe.

### Case presentation

In 2016 a flock of ~ 850 ewes were moved to new dykes at the mouth of the river Elbe in Lower Saxony, district of Cuxhaven, Germany. The area had recently been grazed by another sheep flock with unknown footrot status. The flock under investigation had a history of footrot and the recent flock movement resulted in increased prevalence. This largely closed flock had only had three breeding rams purchased between 2015 and 2018. One ram was purchased from a mixed farm that bred both sheep and cattle whilst the other two were from sheep farms. The footrot status of these three farms and the BDD status of the mixed farm were unknown. Heavy rainfall with warm and humid conditions in the area were recorded in the summer/autumn of 2017, which was a challenge to sheep farming generally. In the flock described, the number of lame sheep increased during this period. Due to persistent wet weather, the whole flock was housed 7 weeks earlier than usual (1st November 2017) in a new-built barn instead of typical transfer to housing at the end of December (prior to lambing).

In December 2017, a local veterinary surgeon asked for advice concerning treatment of 200 lame sheep with severe footrot. The farmer had treated the sheep by claw trimming, footbaths (zinc sulphate) and a topical oxytetracycline spray (Animedazon^®^, Livisto, Senden-Bösensell, Germany), which was the classical footrot treatment [25], but was still unable to stop the spread of lameness in the flock. Since footrot was suspected, advice was given to stop footbathing and to administer gamithromycin (Zactran^®^, Boehringer Ingelheim Vetmedica GmbH, Ingelheim, Germany) or tulathromycin (Draxxin 100 mg/ml ^®^, Zoetis Deutschland GmbH, Berlin, Germany) by injection to all lame sheep. The veterinarian was also advised to avoid claw trimming; claws were therefore only clipped if excessively long, but not earlier than five to 10 days after systemic antibiotic treatment.

About 150 of the severely lame sheep were treated in the first week of January 2018 with gamithromycin. The systemic antibiotic treatment was successful in treating symptomatic individuals within the flock during the housing period, although further asymptomatic, untreated sheep continued to become lame.

On January 15, 2018, the farmer brought two severely lame (locomotion score 5 of 6) sheep suffering from claw inflammation to the University of Veterinary Medicine Hannover. The animals were hospitalized for further examinations, diagnosis and treatment. On January 31, 2018, the farm was visited for clinical examination and sampling of lame sheep.

**Fig. 1:**
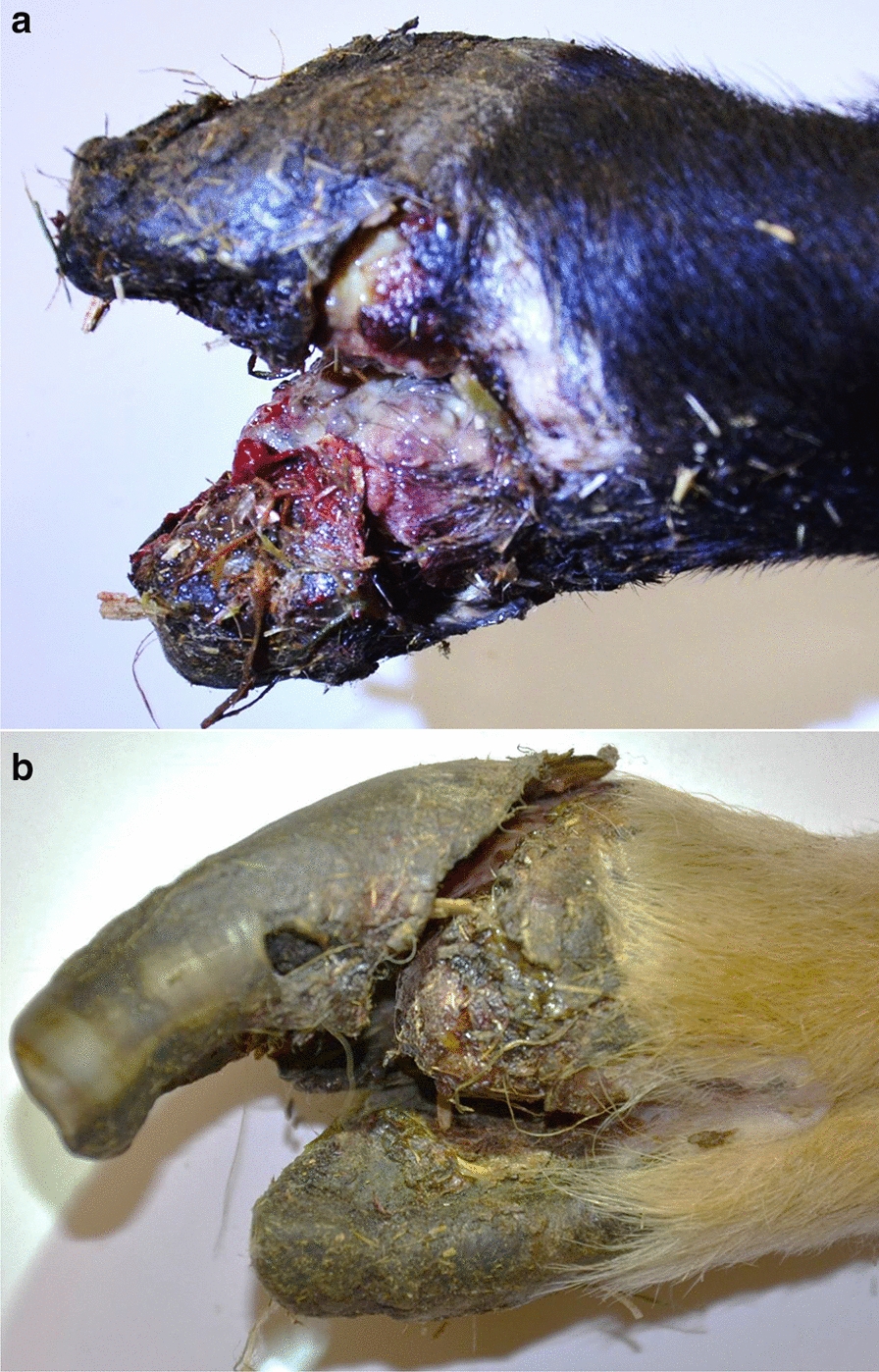
Gross lesions in sheep affected by contagious ovine digital dermatitis. **a** The foot of sheep 1 with detachment of both claw capsules. **b** The foot of sheep 2 with the detachment of the medial claw of the right foreleg

The two hospitalized sheep exhibited severe hoof horn defects, beginning at the proximal coronary band. In both sheep, complete detachment of the hoof horn capsule was observed, which for sheep 1 was present in both claws from the same leg (Fig. 1a) whilst in sheep 2 only one claw was affected (Fig. 1b).

The sheep at the farm were housed with less than one m^2^ per sheep, therefore individual lameness scoring was not possible. The lambs, with a maximum age of 36 days, were not yet affected at that time. Clinical examination of 10 pre-selected lame, untreated sheep revealed severe claw lesions. The loss of horn at the proximal coronary band was prevalent in these animals. During the visit no more newly lame and untreated sheep were found. Details of the clinical findings in the 12 sheep were documented according to a recognised scheme [26] and are listed in additional file 1.

Additionally, biopsies of the coronary band were taken and stored in liquid nitrogen from 11 affected sheep on farm and 10 asymptomatic control sheep from the healthy flock of the Clinic. Tissue samples were taken by skin biopsy stamp (Biopsie Punch Hautstanzen, 3 mm, WDT Curavet, Garbsen, Germany) at the boundary of claw and coronary band and crossed the border between affected/non-affected tissues. Biopsies were taken under local anaesthesia by injecting 1 mL of Procaine-HCl locally subcutaneously (Isocain, Selectavet,Weyarn, Germany) and stored in liquid nitrogen immediately after sampling.

From the two symptomatic, hospitalized sheep, swabs from the interdigital space and coronary band were Gram-stained and microscopically surveyed for spirochaetes. From the 10 pre-selected lame sheep on farm, interdigital swabs were taken for bacterial culture to detect *D. nodosus*. Pus and necrotic material from underneath lost hoof horn and from the brim of infected tissue were collected with bacterial plastic loops and inoculated onto Eugon agar containing 0.2% yeast extract and 10% horse blood with and without 1 µg/mL lincomycin [27]. On farm, streaked culture plates were immediately placed into anaerobic jars with anaerobic gas generating sachets and, in the laboratory of the Institute for Microbiology, incubated at 37 °C for 6 days. Bacteria with characteristic colony morphology were tested using Polymerase Chain Reaction (PCR) [28].

Swabs from the interdigital space and from lesions at the coronary band of the affected feet of the 12 sheep were investigated for *D. nodosus by* PCR [29].

Frozen samples were sent on dry ice to the Department of Infection Biology & Microbiomes, University of Liverpool, United Kingdom, for a molecular genetic examination focussed on *Treponema* spp.. One affected animal with chronic lesions and detachment of hoof horn was not tested for treponemes due to the late stage of the disease. Upon receipt, tissues from lesions and healthy controls were thawed and DNA extracted using a DNeasy Kit (Qiagen, United Kingdom) in accordance with the manufacturer’s instructions. Extracted DNA was stored at − 20 °C until analysis. Samples were subjected to a *Treponema* genus PCR, capable of detecting both pathogenic and commensal *Treponema* spp., as described previously [30]. Samples were additionally subjected to nested PCR assays specific for the three BDD-associated treponeme groups (*T. medium* phylogroup, *T. phagedenis* phylogroup and *T. pedis*) as described previously [10]. A water control and genomic DNA from each of the three treponeme phylogroups were used as negative and positive controls, respectively.

Eleven of the 12 examined sheep showed coronary band lesions and in 6 of 12, one horn capsule was detached (Additional file 1). Typically, coronary band lesions and exungulations were found in all sheep and involved at least one foot. In addition, typical footrot symptoms with inflammation running from the interdigital space under the axial horn to the sole followed by an inflammation of the abaxial dermis were found in all 12 sheep, with 8 of the 12 having more than one foot affected.

Gram staining of claw swab smears from the symptomatic sheep in the clinic identified spirochaetes on microscopic examination (Table 1). *D. nodosus* could be isolated and differentiated from 2 of the 10 sheep sampled on farm (Table 1). The *D. nodosus* PCR was negative for one hospitalized sheep with an inconclusive result for the second (Table 1). Claw swab samples of the 10 sheep sampled in the flock were positive in seven cases with a further two samples inconclusive.

**Table 1:** Findings in claw swabs or claw biopsies by different bacteriological methods

Animal no.	Sampling at	TWDS-Score	Detection of Spirochaetes by Gram stain	*D. nodosus* culture	*D. nodosus* PCR	Treponema spp. PCR	T. medium/T. vincentii-like	T. phagedenis-like	T. pedis
1	Clinic	15	+	n.d.	–	+	+	+	+
2	Clinic	18	+	n.d.	±	+	+	+	+
3	Farm	34	n.d.	–	+	+	+	+	+
4	Farm	72	n.d.	–	+	+	+	+	+
5	Farm	102	n.d.	–	±	+	+	+	+
6	Farm	52	n.d.	–	±	+	+	+	+
7	Farm	36	n.d.	–	±	n.d.	n.d.	n.d.	n.d.
8	Farm	40	n.d.	–	+	+	+	+	+
9	Farm	4	n.d.	–	+	+	+	+	+
10	Farm	34	n.d.	+	+	+	+	+	+
11	Farm	136	n.d.	+	+	+	+	+	+
12	Farm	68	n.d.	–	+	+	–	+	+
13	Control	0	n.d.	n.d.	n.d.	–	–	–	–
14	Control	0	n.d.	n.d.	n.d.	+	+	–	+
15	Control	0	n.d.	n.d.	n.d.	+	–	–	–
16	Control	0	n.d.	n.d.	n.d.	+	–	–	–
17	Control	0	n.d.	n.d.	n.d.	–	–	–	–
18	Control	0	n.d.	n.d.	n.d.	+	–	–	–

+, positive; - , negative; ±, inconclusive; TWDS, Total weighted digital score according to Wittington and Nicholls (1995) [41]; n.d., not determined

In all 10 clinically symptomatic samples *Treponema* spp. PCR and specific PCRs for *T. medium* phylogoup*, T. phagedenis* phylogroup and *T. pedis* were positive (Table 1). One sample from a healthy control sheep was positive for *T. medium* and *T. pedis* in the treponeme phylogroup-specific PCR assay also.

## Discussion and conclusions

In the flock described here, the conventional footrot treatments with hoof trimming and footbaths failed. As spirochaete bacteria were initially detected in the two hospitalized cases by Gram-staining of the smears of the interdigital space, diagnosis of CODD was suspected, although footrot was not excluded [31], for *D. nodosus* was detected by culture and PCR in some of the affected sheep (see Table 1). The diagnosis of CODD was confirmed by identifying specific, pathogenic *Treponema* phylogroups in all 11 sheep tested by PCR. In all 11 sheep, *T. pedis* and *T. phagedenis* phylogroup were present, with *T. medium* phylogroup identified in 10 of the 11. This is the first published report of CODD in sheep in Germany and in mainland Europe.

As previously reported, the genus-specific *Treponema* assay is not disease specific [13], and our tests showed positive results for both affected and one healthy animal. This control animal was tested positive for the CODD specific phylogroups *T. medium* and *T. pedis* by PCR. The control sheep are housed in the direct vicinity of pigs, whose gastrointestinal tract can be a source of commensal treponemes as well as *Treponema pedis* [32, 33]. Up to now (July 2020), none of the control sheep, which are still housed on the same location, have developed lameness.

The flock where CODD occurred had a long history of footrot. The prevalence of lameness in the flock was estimated to be less than 5% in the summer of 2017 by the farmer. However, due to the endemic situation of BDD in the cattle herds in that region, there were potentially numerous possibilities for treponemal infections to spread between infected cattle and naive sheep flocks. A ram introduced from a mixed sheep and cattle farm is one possible infection source. Current evidence suggests potential roles for the bovine gastrointestinal tract, slurry, and hoof trimming equipment in the transmission of BDD [33–36]. It is very common in the northern coastal region of Germany for sheep to graze in late autumn and winter on pastures that have been previously used for grazing cattle, or fertilised by slurry. BDD is highly prevalent in the cattle herds in that region [37], and this may represent an important disease transmission route [38]. Introduction of CODD might also have been caused by visitors to the dyke or the farm. Hoof trimming was only performed by the shepherd and his son; mitigating any infection risk posed from professional hoof trimmers [35].

As most sheep flocks in Germany are closed epidemiological units, it should be possible to control the spread of CODD between sheep farms. In general, only breeding rams are purchased, and it is therefore possible that the quarantine of rams could help to avoid introduction of CODD. In Germany, no stratification system exists. The presence of BDD in cattle on farm is a risk factor for CODD [39], suggesting that if sheep are co-grazed there may be transmission of BDD treponemes. Furthermore, given the intestinal tract of cattle is a known reservoir of BDD treponemes [34], there may be a potential infection risk if sheep are grazed on pastures fertilised with cattle slurry. There was presumably direct or indirect contact of the affected flock to dairy cattle with BDD. Whilst sheep were not purchased from other farms, several other reported risk factors including large flock size, adult sheep, time of year and the housing of sheep [39, 40] were identified for this flock. These risk factors might have increased the susceptibility of this flock to footrot and CODD. Moreover, the summer and autumn months of 2017 were warm with unusually high rainfall. Even in the winter of 2017/18, pastures were wet and the feet of these sheep were exposed to prolonged wet conditions. The affected animals were housed in the new barn, but there were no other means adopted to stop the distribution of infection other than classical footrot treatment. Despite treatment failure, affected animals were not isolated and the barn was not mucked, or disinfected to reduce risk of transmission. Neither the local veterinarian nor the farmer were previously aware of CODD.

In summary we present the first recorded case of CODD in mainland Europe and we encourage continued vigilance and surveillance of CODD both in Europe and worldwide.

## Supplementary information

**Additional file 1:** Clinical findings at the claws with footrot scoring according to Egerton and Roberts (1971) and additional findings. *TWDS = Total weighted digital score according to Whittington und Nicholls [41]. **CODD lesion grade according to Angell et al. [5].

## Data Availability

The datasets used are available from the corresponding author on reasonable request.
